# Identification of Prognostic Factors Related to Morbidity, Mortality, and Increased Healthcare Expenditure Following Surgery for Femoral Fracture or Hip Arthroplasty

**DOI:** 10.7759/cureus.84056

**Published:** 2025-05-13

**Authors:** Zachary A Blashinsky, Silas Helbig, Chrisnel Lamy, Noel C Barengo, Rupa Seetharamaiah, Juan Ruiz-Pelaez

**Affiliations:** 1 Orthopedic Surgery, Florida International University, Herbert Wertheim College of Medicine, Miami, USA; 2 Health Sciences, Florida International University, Herbert Wertheim College of Medicine, Miami, USA; 3 Epidemiology and Biostatistics, Florida International University, Herbert Wertheim College of Medicine, Miami, USA; 4 Medicine, Riga Stradiņš University, Riga, LVA; 5 Global Health, Florida International University, Robert Stempel College of Public Health, Miami, USA; 6 Translational Medicine, Florida International University, Herbert Wertheim College of Medicine, Miami, USA; 7 Surgery, Florida International University, Herbert Wertheim College of Medicine, Miami, USA; 8 Surgery, Baptist Hospital, Miami, USA; 9 Medical Education, Kangaroo Foundation, Bogota, COL

**Keywords:** femoral fracture, orthopedic surgery, postoperative complications, statistics and numerical data, surgical risk factors, total hip arthroplasty, value based care, length of stay

## Abstract

Introduction

Postoperative outcomes following hip arthroplasty and femoral fracture surgeries significantly impact patient care and healthcare resources. This study aimed to identify modifiable and non-modifiable prognostic factors that independently predict major postoperative complications and increased hospital resource utilization in these patients.

Methods

We conducted a retrospective cohort study using the 2019 National Surgical Quality Improvement Program (NSQIP) database, including adult patients who underwent hip arthroplasty or femoral fracture treatment. Patients with incomplete data were excluded. The primary outcome was a composite of major adverse events, including mortality and 11 complications; the secondary outcome was healthcare resource utilization, assessed by length of stay and readmissions. We used stepwise backward multivariable logistic regression for analysis.

Results

Out of 176,801 cases, 12,146 (6.87%) experienced adverse outcomes. Significant predictors of adverse events included higher American Society of Anesthesiologists (ASA) classification, age ≥65 years, underweight body mass index (BMI), male sex, use of general anesthesia, and comorbidities such as COPD, insulin-dependent diabetes, ascites, congestive heart failure (CHF), hypertension, dialysis requirement, steroid use, bleeding disorders, and sepsis. Overweight and obese BMI were protective against adverse events. Increased resource utilization was associated with higher ASA classification, underweight BMI, use of general anesthesia, and comorbidities like insulin and non-insulin-dependent diabetes, COPD, CHF, hypertension, dialysis, steroid use, bleeding disorders, and SIRS. Again, overweight and obese BMIs were protective. The predictive model achieved a mean area under the curve (AUC) of 0.73 through 10-fold cross-validation.

Conclusions

Key predictors of adverse outcomes and increased hospital resource use include specific comorbidities and surgical factors, notably underweight BMI and higher ASA classification. Targeted interventions to optimize perioperative care for high-risk patients are necessary to minimize complications. These findings can guide clinical practice and surgical decision-making. Further research should explore these associations and refine preoperative risk stratification models.

## Introduction

Postoperative outcomes of complex orthopedic procedures, such as hip arthroplasty and treatment for femoral fractures, are critical benchmarks for both surgical performance and patient well-being [1]. Surgical complications, along with increased morbidity and mortality, have additional associated costs and burden on the healthcare system. As medical and technological advancements shape the field, there is an increasing emphasis on providing value to patients by reducing healthcare costs and optimizing resource allocation [2]. This requires a comprehensive understanding of the relationship between patients' prognostic factors and potential postoperative complications. Using the American College of Surgeons National Surgical Quality Improvement Program (ACS-NSQIP) database, a large nationwide dataset, this research aims to identify and quantify the independent association of 16 preoperative prognostic factors with a composite outcome of morbidity, mortality, and surgical complications, as well as secondary outcomes such as hospital resource utilization (e.g., length of stay, readmission, and reintervention). By identifying these factors, the study seeks to improve surgical planning, patient selection, and targeted prophylactic measures, minimizing complications and optimizing recovery. The findings will enhance prognostic understanding and inform tailored interventions, empowering patients and physicians with accurate information and advancing personalized care in orthopedic surgery.

## Materials and methods

This is a secondary data analysis in which we used data from the NSQIP registry from the years 2018 and 2019 to conduct a historical cohort study with an exploratory analysis. We chose to use 2019 and earlier to avoid the effects of the COVID-19 pandemic on the study. The ACS-NSQIP is a nationally validated, risk-adjusted, outcomes-based database used by hospitals throughout the United States to measure and improve the quality of surgical care. It employs a prospective, peer-controlled, validated database to quantify 30-day, risk-adjusted surgical outcomes, which validly compares outcomes among all hospitals in the program. Peer-reviewed studies have shown that ACS NSQIP effectively improves the quality of surgical care while reducing complications and costs.

The study population consisted of adult patients who underwent surgery for hip arthroplasty or surgical treatment of a femoral fracture, as identified by the following Current Procedural Terminology (CPT) codes: 27125 (hemiarthroplasty, hip, partial, e.g., femoral stem prosthesis, bipolar arthroplasty), 27130 (total hip arthroplasty (THA)), 27132 (conversion of previous hip surgery to total hip arthroplasty (conversion THA)), 27236 (open treatment of proximal femoral fracture, e.g., femoral neck, intertrochanteric, or subtrochanteric, with internal fixation), and 27269 (open treatment of proximal femoral fracture with prosthetic replacement). Patients in the database with missing data on any of the independent variables or the outcomes were excluded.

The potential prognostic factors explored included age, body mass index (BMI), gender, smoking status, American Society of Anesthesiologists (ASA) classification, anesthesia technique, COPD, ascites, heart failure within 30 days prior to surgery, hypertension requiring medication, need for preoperative dialysis, use of immunosuppressive therapy, bleeding disorder, and sepsis before operation.

The primary composite outcome consisted of postoperative death or the occurrence of at least one of the following complications within 30 days after surgery: cardiac arrest, myocardial infarction, CVA/stroke, pulmonary embolism (PE), deep vein thrombosis (DVT), septic shock, sepsis, pneumonia, wound dehiscence, organ space infection, or deep incisional surgical site infection. The secondary composite outcome focused on hospital resource utilization, including any readmission, unplanned reoperation, unplanned reintubation, and hospital length of stay exceeding 30 days.

An exploratory analysis was conducted to describe the distribution of variables. A bivariate analysis was performed to examine the distribution of the outcomes according to each potential predictor and assessed for statistical significance using a chi-square or Fisher's exact test, as appropriate. Continuous variables (age and BMI) were categorized. Collinearity among potential predictors was assessed using Spearman correlation coefficients. A binary unconditional logistic regression model was then fitted, including all non-collinear predictors. The initial saturated model was subsequently reduced to the most parsimonious set of predictors that best predicted the outcome, using a stepwise backward elimination algorithm.

## Results

After applying the inclusion criteria, a total of 176,801 cases were included in the study sample. Table 1 outlines the occurrence of adverse events among the study population. The most frequent complication was death, reported in 1.39% of cases, followed closely by pneumonia at 1.14%. Wound infections accounted for 0.94% of complications, while myocardial infarction and DVT were observed in 0.62% and 0.46% of participants, respectively. Less common events included sepsis (0.44%), PE (0.41%), and organ space surgical site infections (0.40%). Rare events, such as septic shock and wound disruption, occurred in fewer than 0.20% of cases.

**Table 1:** Frequency of adverse events DVT, deep vein thrombosis

Complication	Frequency (Cases)	Percentage (%)
Death	2,452	1.39
Pneumonia	2,012	1.14
Wound Infection	1,663	0.94
Myocardial Infarction	1,100	0.62
DVT	815	0.46
Sepsis	774	0.44
Pulmonary Embolism	732	0.41
Organ Space Surgical Site Infection	700	0.40
Unplanned Intubation	538	0.30
Stroke/CVA	405	0.23
Cardiac Arrest	357	0.20
Septic Shock	316	0.18
Wound Disruption	282	0.16

The distribution of the incidence of the primary composite outcome is presented in Table 2. Seniors, defined as those aged 65 and older, showed the highest incidence of adverse events, with 6.70% of this group affected, more than double compared to the younger age categories. Similarly, underweight individuals (BMI <18.5) were at elevated risk, with 14.32% experiencing complications. In contrast, a healthy BMI (18.5-24.9) had the lowest rate of adverse events. Furthermore, patients with insulin-dependent diabetes, severe COPD, and those who received general anesthesia had significantly higher rates of complications. Notably, 6.84% of patients undergoing general anesthesia experienced an adverse event, compared to 3.40% in those receiving spinal anesthesia.

**Table 2:** Incidence of adverse events according to patients’ characteristics P<0.05 is considered statistically significant. BMI, body mass index; CHF, congestive heart failure; ASA, American Society of Anesthesiologists

Characteristics	No Adverse Event (n)	No Adverse Event (%)	Adverse Event (n)	Adverse Event (%)	Total (n)	p-value
Age Category						<0.001
Young Adult	2,020	97.40%	54	2.60%	2,074	-
Middle Aged	10,362	98.21%	189	1.79%	10,551	-
Advanced Age	48,702	97.91%	1,041	2.09%	49,743	-
Senior	106,768	93.30%	7,665	6.70%	114,433	-
Smoking Status	-	-	-	-	-	0.693
Non-Smoker	147,977	94.95%	7,877	5.05%	155,854	-
Smoker	19,875	94.88%	1,072	5.12%	20,947	-
BMI Category	-	-	-	-	-	<0.001
Underweight	5,641	85.68%	943	14.32%	6,584	-
Healthy	38,528	92.82%	2,979	7.18%	41,507	-
Overweight	51,742	96.03%	2,138	3.97%	53,880	-
Obese	66,121	96.83%	2,168	3.17%	68,289	-
Diabetes Status	-	-	-	-	-	<0.001
Insulin-Dependent	6,337	88.90%	791	11.10%	7,128	-
Non-Insulin-Dependent	16,103	94.10%	1,009	5.90%	17,112	-
No Diabetes	145,412	95.31%	7,149	4.69%	152,561	-
Sex	-	-	-	-	-	<0.001
Female	97,180	95.15%	4,956	4.85%	102,136	-
Male	70,666	94.65%	3,993	5.35%	74,659	-
Anesthesia Type	-	-	-	-	-	<0.001
General Anesthesia	81,150	93.16%	5,957	6.84%	87,107	-
MAC/IV	27,954	96.78%	930	3.22%	28,884	-
Spinal	56,051	96.60%	1,972	3.40%	58,023	-
COPD History	-	-	-	-	-	<0.001
No COPD	159,610	95.44%	7,618	4.56%	167,228	-
Severe COPD	8,242	86.10%	1,331	13.90%	9,573	-
Ascites	-	-	-	-	-	<0.001
No Ascites	167,770	94.96%	8,906	5.04%	176,676	-
Yes Ascites	82	65.60%	43	34.40%	125	-
CHF	-	-	-	-	-	<0.001
No CHF	166,136	95.20%	8,371	4.80%	174,507	-
Yes CHF	1,716	74.80%	578	25.20%	2,294	-
Hypertension	-	-	-	-	-	<0.001
No Hypertension	73,420	96.30%	2,820	3.70%	76,240	-
Hypertension	94,432	93.91%	6,129	6.09%	100,561	-
Dialysis	-	-	-	-	-	<0.001
No Dialysis	166,985	95.03%	8,742	4.97%	175,727	-
Yes Dialysis	867	80.73%	207	19.27%	1,074	-
Steroid Use	-	-	-	-	-	<0.001
No Steroid Use	161,408	95.11%	8,296	4.89%	169,704	-
Steroid Use	6,444	90.80%	653	9.20%	7,097	-
Bleeding Disorder	-	-	-	-	-	<0.001
No Bleeding Disorder	160,718	95.49%	7,596	4.51%	168,314	-
Bleeding Disorder	7,134	84.06%	1,353	15.94%	8,487	-
Sepsis						<0.001
No Sepsis	163,297	95.48%	7,723	4.52%	171,020	-
SIRS	4,383	79.98%	1,097	20.02%	5,480	-
Sepsis	160	59.04%	111	40.96%	271	-
Septic Shock	12	40.00%	18	60.00%	30	-
ASA Classification	-	-	-	-	-	<0.001
No Disturbance	4,692	99.24%	36	0.76%	4,728	-
Mild Disturbance	73,453	98.28%	1,283	1.72%	74,736	-
Severe Disturbance	80,218	93.88%	5,233	6.12%	85,451	-
Life Threatening	9,209	79.64%	2,354	20.36%	11,563	-
Moribund	57	71.25%	23	28.75%	80	-

Table 3 presents the unadjusted and adjusted odds ratios (aORs) for potential predictors of adverse events. Age, BMI, and ASA classification had the strongest association with the outcome. Patients classified as seniors had nearly twice the odds of experiencing an adverse event compared to middle-aged individuals, with an aOR of 1.90 (95% CI 1.61-2.26). Similarly, being underweight was associated with a 54% higher likelihood of complications (aOR 1.54, 95% CI 1.40-1.69). On the other hand, insulin-dependent diabetes significantly increased the odds of adverse events, with an aOR of 1.53 (95% CI 1.38-1.68). Interestingly, being overweight or obese appeared to confer a protective effect, with odds of adverse events reduced by 34% and 45%, respectively (overweight: aOR 0.66, 95% CI 0.61-0.70; obese: aOR 0.55, 95% CI 0.51-0.59).

**Table 3:** Adjusted and unadjusted ORs for adverse events according to potential predictors P<0.05 is considered statistically significant. BMI, body mass index; CHF, congestive heart failure; ASA, American Society of Anesthesiologists; ORs, odds ratios

Characteristics	Unadjusted OR (95% CI)	Adjusted OR (95% CI)	p-value
Age Category			
Middle Aged (36-50)	Ref	Ref	-
Young Adult (18-35)	1.50 (1.07-2.11)	1.32 (0.93-1.87)	0.122
Advanced Age (51-64)	1.17 (0.98-1.39)	1.02 (0.85-1.22)	0.835
Senior (65+)	3.87 (3.29-4.55)	1.90 (1.61-2.26)	<0.001
BMI Category	-	-	-
Healthy (18.5-24.9)	Ref	Ref	-
Underweight (0-18.4)	2.14 (1.96-2.34)	1.54 (1.40-1.69)	<0.001
Overweight (25-29.9)	0.54 (0.51-0.58)	0.66 (0.61-0.70)	<0.001
Obese (30.0+)	0.43 (0.40-0.45)	0.55 (0.51-0.59)	<0.001
Diabetes Status	-	-	-
No Diabetes	Ref	Ref	-
Insulin-Dependent	2.52 (2.31-2.75)	1.53 (1.38-1.68)	<0.001
Non-Insulin-Dependent	1.28 (1.19-1.38)	1.08 (0.99-1.17)	0.080
Sex	-	-	-
Female	Ref	Ref	-
Male	1.13 (1.07-1.18)	1.33 (1.26-1.41)	<0.001
Anesthesia Type	-	-	-
Spinal	Ref	Ref	-
General Anesthesia	2.08 (1.96-2.20)	1.58 (1.48-1.69)	<0.001
MAC/IV	0.95 (0.87-1.04)	0.98 (0.89-1.08)	0.682
All Other	1.04 (0.83-1.32)	1.05 (0.82-1.34)	0.717
COPD History	-	-	-
No	Ref	Ref	-
Yes	3.41 (3.18-3.66)	1.64 (1.51-1.77)	<0.001
Ascites	-	-	-
No	Ref	Ref	-
Yes	9.28 (6.11-14.09)	2.58 (1.57-4.25)	<0.001
CHF	-	-	-
No	Ref	Ref	-
Yes	6.75 (6.06-7.52)	2.08 (1.84-2.35)	<0.001
Hypertension	-	-	-
No	Ref	Ref	-
Yes	1.65 (1.57-1.74)	1.07 (1.01-1.14)	0.022
Dialysis	-	-	-
No	Ref	Ref	-
Yes	4.46 (3.75-5.31)	1.40 (1.16-1.71)	<0.001
Steroid Use	-	-	-
No	Ref	Ref	-
Yes	1.97 (1.80-2.17)	1.40 (1.26-1.56)	<0.001
Bleeding Disorder	-	-	-
No	Ref	Ref	-
Yes	3.98 (3.71-4.27)	1.61 (1.48-1.74)	<0.001
Sepsis	-	-	-
No	Ref	Ref	-
SIRS	5.08 (4.69-5.50)	2.28 (2.09-2.49)	<0.001
Sepsis	12.94 (9.83-17.02)	5.24 (3.87-7.09)	<0.001
Septic Shock	26.78 (12.15-59.01)	6.29 (2.53-15.60)	<0.001
ASA Classification	-	-	-
I: No Disturbance	Ref	Ref	-
II: Mild Disturbance	2.16 (1.51-3.09)	1.99 (1.36-2.91)	<0.001
III: Severe Disturbance	8.02 (5.63-11.44)	4.84 (3.32-7.07)	<0.001
IV: Life Threatening	31.08 (21.74-44.41)	10.91 (7.45-15.99)	<0.001
V: Moribund	51.08 (27.20-95.91)	13.25 (6.48-27.09)	<0.001

Next, we calculated the unadjusted ORs and aORs for critical predictors of hospital resource utilization, shown in Table 4. BMI, anesthesia type, and comorbidities such as diabetes, COPD, and congestive heart failure (CHF) were significant factors. Underweight individuals had a 28% greater likelihood of increased resource utilization than those with a healthy BMI (aOR: 1.28, 95% CI: 1.14-1.43). In contrast, being overweight reduced the odds by 24%, while obese patients saw a 30% reduction (aOR: 0.76, 95% CI: 0.70-0.82, and aOR: 0.70, 95% CI: 0.65-0.76, respectively). Insulin-dependent diabetics faced a 36% higher chance of requiring more hospital resources (aOR: 1.36, 95% CI: 1.22-1.51). Similarly, general anesthesia was associated with a 34% increase in odds compared to spinal anesthesia (aOR: 1.34, 95% CI: 1.26-1.44). Several comorbidities were strongly linked to higher resource use. COPD increased the odds by 45% (aOR: 1.45, 95% CI: 1.33-1.59), and CHF was linked to a 51% rise in the likelihood of greater resource utilization (aOR: 1.51, 95% CI: 1.30-1.75). Dialysis patients had more than double the odds of requiring additional hospital resources (aOR: 2.04, 95% CI: 1.67-2.48). ASA classification severity further influenced outcomes, with those in ASA class IV (life-threatening conditions) experiencing a 4.67-fold increase in odds (95% CI: 3.49-6.24), and patients classified as moribund (ASA class V) also had over four times the likelihood (aOR: 4.15, 95% CI: 1.77-9.74) compared to patients with no disturbance (ASA class I).

**Table 4:** ORs for hospital resource utilization according to each potential predictor P<0.05 is considered statistically significant. BMI, body mass index; CHF, congestive heart failure; ASA, American Society of Anesthesiologists; ORs, odds ratios

Predictor	Unadjusted OR (95% CI)	Adjusted OR (95% CI)	p-value
BMI Category			
Healthy (18.5-24.9)	Ref	Ref	-
Underweight (<18.5)	1.38 (1.23-1.54)	1.28 (1.14-1.43)	<0.001
Overweight (25-29.9)	0.71 (0.66-0.77)	0.76 (0.70-0.82)	<0.001
Obese (≥30)	0.65 (0.61-0.70)	0.70 (0.65-0.76)	<0.001
Diabetes Status	-	-	-
No Diabetes	Ref	Ref	-
Insulin-Dependent	1.58 (1.43-1.75)	1.36 (1.22-1.51)	<0.001
Non-Insulin-Dependent	1.12 (1.03-1.21)	1.14 (1.04-1.25)	0.004
Anesthesia Type	-	-	-
Spinal	Ref	Ref	-
General Anesthesia	1.48 (1.39-1.58)	1.34 (1.26-1.44)	<0.001
COPD History	-	-	-
No COPD	Ref	Ref	-
COPD	1.82 (1.68-1.98)	1.45 (1.33-1.59)	<0.001
CHF	-	-	-
No CHF	Ref	Ref	-
CHF	2.46 (2.14-2.81)	1.51 (1.30-1.75)	<0.001
Hypertension	-	-	-
No Hypertension	Ref	Ref	-
Hypertension	1.34 (1.26-1.43)	1.20 (1.12-1.28)	<0.001
Dialysis	-	-	-
No Dialysis	Ref	Ref	-
Dialysis	3.43 (2.86-4.11)	2.04 (1.67-2.48)	<0.001
Steroid Use	-	-	-
No Steroid Use	Ref	Ref	-
Steroid Use	1.59 (1.43-1.78)	1.41 (1.25-1.58)	<0.001
Bleeding Disorder	-	-	-
No Bleeding Disorder	Ref	Ref	-
Bleeding Disorder	1.73 (1.58-1.88)	1.28 (1.16-1.40)	<0.001
Sepsis Status	-	-	-
No Sepsis	Ref	Ref	-
SIRS	1.99 (1.80-2.21)	1.44 (1.29-1.61)	<0.001
ASA Classification	-	-	-
I: No Disturbance	Ref	Ref	-
III: Severe Disturbance	3.97 (3.02-5.20)	2.90 (2.19-3.84)	<0.001
IV: Life-Threatening	8.72 (6.61-11.50)	4.67 (3.49-6.24)	<0.001
V: Moribund	9.15 (4.29-19.53)	4.15 (1.77-9.74)	<0.001

Finally, as evaluated through a 10-fold cross-validation method, the model's predictive performance achieved a mean area under the curve (AUC) of 0.73, indicating a moderate ability to discriminate between patients with and without adverse events. The AUC scores across the 10 folds ranged from a high of 0.76 to a low of 0.69. The Hosmer-Lemeshow test confirmed that the model fit was appropriate, further validating the robustness of the predictions. Table 5 illustrates an analysis of the model, which showed a cross-validated mean AUC of 0.73, a bootstrap bias-corrected of 0.72 to 0.74 (95% CI), and a cross-validated standard deviation of 0.0207. 

**Table 5:** AUC results from cross-validation AUC, area under the curve

Fold	Number of Observations (N)	AUC
1-fold	4,329	0.735
2-fold	4,329	0.715
3-fold	4,328	0.693
4-fold	4,329	0.755
5-fold	4,328	0.715
6-fold	4,329	0.724
7-fold	4,329	0.738
8-fold	4,328	0.744
9-fold	4,329	0.758
10-fold	4,328	0.712

## Discussion

Our predictive model demonstrated that patients with severe ASA classification and sepsis had the greatest likelihood of developing the adverse events identified. The analysis identified several critical factors associated with both adverse events and increased hospital resource utilization in patients undergoing surgical procedures. For adverse events, age, BMI, diabetes status, ASA classification, and chronic health conditions (e.g., COPD, heart failure, sepsis) were found to be statistically significant predictors. The results align with clinical expectations, as patients with worse baseline health were at higher risk for complications.

Most studies focused on specific prognostic factors for functional recovery rather than the occurrence of adverse events and the utilization of hospital resources, indicating a gap in the literature that deserves further investigation [3-6]. Meanwhile, our study looked at an aggregate of potential confounders and evaluated the OR of a composite variable of several adverse outcomes on patients undergoing THA and femoral fixation. This allows for the analysis of covariates and provides a predictive model as unique as each patient. 

Hewlett-Smith et al. systematically reviewed prognostic factors for inpatient functional recovery following total hip and knee arthroplasties [6]. There was conflicting evidence for BMI and age as prognostic factors in both populations. The role of sex was supported only by limited evidence in total knee arthroplasty (TKA) and conflicting evidence in THA studies. This review did not identify any independently prognostic surgical factors for postoperative functional recovery. Van der Sijp et al. systematically reviewed independent factors associated with functional outcomes in patients with proximal femoral fractures [5]. Comorbidities, age, cognition, and functionality were factors for which most studies indicated a significant effect. The factors identified showed a significant correlation with short-term functional outcomes and mortality. Sniderman et al. used a machine learning approach to build a predictive model using patient-specific variables and their effect on postoperative functional outcomes in THA [4]. The variables that predicted a worse outcome in order of strength of association consisted of frequent thoughts of work, frequent comparison to healthier peers, increased BMI, increased medical comorbidities, and the anterior surgical approach. Kee et al. conducted a retrospective review of all revision total hip and knee arthroplasties at a single center within two years of primary surgery [3]. Patients with infected hip revision surgery were more likely to have increased BMI than those without infection. Patients with infected knee revision were more likely to smoke, have poor dentition, and have more than one contraindication compared to patients without infection. They concluded that a high percentage of patients undergoing early revision arthroplasty had modifiable risk factors before undergoing primary joint arthroplasty. Rojas-García et al. systematically reviewed the impacts and costs of delayed discharge [7]. They concluded that the majority of research in the area is of poor quality. Nine of the studies linked smoking to adverse outcomes, including increased length of stay, complications, and mortality [8-16]. Moller et al. was the only pertinent randomized control trial found during the literature review [15]. They studied the effect of preoperative smoking intervention in patients undergoing hip and knee arthroplasty on complication rate. The complication rate was 18% in the group who underwent smoking intervention and 52% in the control group (p=0.0003). Otero et al. studied complications and readmission among THA, TKA, and unicompartmental knee arthroplasty (UKA) [17]. Smoking was associated with increased complication or readmission within 30 days, with an OR of 1.3 (1.2-1.5) for THA and TKA and 2.1 (1.0-4.3) for UKA. However, a direct relationship between length of stay and smoking was not studied. While many studies have articulated the increased risk for surgical complications and poorer outcomes in patients who smoke [8-14,18,19], our study did not demonstrate this based on the information available on the dataset utilized. Smoking did not show a statistically significant difference in the rate of adverse events in our patient population compared to non-smokers. This may be due to some inadequacies of the dataset, particularly the dichotomous nature of smoking in which the information was presented. The NSQIP asked “Had you smoked within the last year?” followed by a yes or no response. This fails to show the dose-dependent nature of smoking and will exclude patients with significant, chronic smoking exposure who have ceased so in the last year.

Liu et al. analyzed the Premier Healthcare Database for risk factors associated with thromboembolic complications after THA [20]. They identified 544,298 THAs between 2015 and 2021, with 0.21% and 0.33% developing PE and DVT, respectively. Chronic pulmonary disease (aOR: 1.58), pulmonary hypertension (aOR: 2.06), and history of VTE (aOR: 2.38) were all comorbidities associated with increased risk of PE. Additionally, age and Black race were non-modifiable risk factors for the development of PE. A French retrospective cohort study from a large single institution analyzed modifiable and non-modifiable factors associated with increased length of stay following THA or TKA [21]. They found factors predicting discharge to rehabilitation centers were older age, female gender, COPD, anxiety-depressive disorder, and stroke history. They also reviewed risk factors for 30-day readmissions, including male gender, obesity, and discharge to rehabilitation centers. 

Rozell et al. created a model to identify comorbidities associated with increased risk of major complications [22]. They analyzed 802 patients who underwent elective primary THA and TKA over nine months. Logistic regression models showed an increased risk of complication in patients with a history of cirrhosis, CHF, and chronic kidney disease.

Focusing on the economic impact of hospital readmissions, Kurtz et al. reported that 30- and 90-day readmissions after THA were 4% and 8%, respectively [23]. They also discovered that 59% of total readmission costs to the US healthcare system were procedure-related complications including infections, dislocations, and periprosthetic fractures. This study emphasizes the need to determine proactively a patient’s candidacy to undergo such surgeries. By inappropriately or completely failing to weigh the cost-benefit, we open each patient up to a myriad of potential complications and additional cost burdens onto our healthcare system.

Courtney et al. compared complication rates among outpatient and inpatient groups and performed a multivariate logistic regression analysis to identify risk factors for poor outcomes [24]. Utilizing the ACS NSQIP database on patients who underwent primary TKA or THA from 2011 to 2014, they reported that outpatient and inpatient groups had complication rates of 8% and 16%, respectively. Additionally, patients greater than 70 years old with malnourishment, cardiac history, smoking history, and diabetes mellitus were associated with a higher risk for readmission and complications. They argue that outpatient treatment may be a superior option for specific, healthy patients who lack the risk factors.

With a variety of comorbidities, it may sometimes be challenging to make a clinical decision whether to undergo surgery. The clinician must make a judgment call to determine the best course of action for the patient: perform the surgery to repair the injury or defer to non-operative management. A patient’s unique characteristics and medical conditions may affect the outcome of the surgery and may increase the incidence of adverse outcomes, including increased morbidity and mortality. Our predictive model will allow clinicians to make informed decisions and explain their chances of developing adverse outcomes to patients. A 1996 study analyzing 6301 surgical procedures showed that ASA classification strongly predicted perioperative complications, postoperative complications, and mortality [16]. Perioperative complications included blood loss, duration of ventilation, and ICU stay. The ASA physical status classification system was designed to offer clinicians an objective metric to properly quantify a patient’s surgical risk [25]. Independent analyses have demonstrated ASA class as a reliable predictor for postoperative complications and mortality across procedures [26]. Although the system provides valuable data, many argue against its inconsistent use across providers and institutions [27]. Sepsis and prosthetic joint infection (PJI) are of paramount concern for orthopedic surgeons. Mortality rates are approximately 20%; however, the exact etiology remains unknown [28]. It is likely due to co-morbidities associated with other chronic conditions. Drain et al. reported that the three most frequent causes of death were respiratory failure, cancer, and renal failure in both aseptic and septic cohorts who underwent TKA [28].

The secondary outcome analysis revealed that predictors such as insulin-dependent diabetes, ASA classification, dialysis, and underweight were also strongly associated with higher hospital resource utilization. These results underscore the importance of preoperative risk assessment, as patients with these risk factors may require more intensive postoperative care. Interestingly, BMI categories showed that underweight patients had an increased risk of adverse events and increased hospital resource use. In contrast, overweight and obese patients had lower odds of adverse events and hospital resource utilization. BMI has come under scrutiny for its ability to estimate body fat and has a poor capability of differentiating muscle mass from fat mass. Previous studies have been inconsistent concerning BMI as a predictor of increased hospital resource utilization. Harvey et al. found that a BMI categorized as obese (>29.9) was associated with increased rates of reintubation after hip arthroplasty surgery [29]. Papalia et al. found no association between BMI and hospital length of stay after THA [30]. These findings suggest that the traditional perception of BMI's role in preoperative assessment for postoperative risk needs further exploration, considering other health factors that interact with BMI, such as metabolic syndrome, diabetes status, serum lipid levels, and patient nutritional status.

This study is intended to be an initial analysis; thus, this model has several limitations. With a mean AUC of 0.729, the model demonstrates only moderate predictive power. This leaves room for improvement, mainly when predicting rare but severe events. Moreover, the ACS NSQIP is an imbalanced dataset. Adverse events were rare in this dataset, with most complications occurring in less than 2% of cases. This imbalance can skew the model's performance, making it more likely to predict non-events. Another factor that may have skewed the results includes the categorization of variables. For ease of analysis, variables such as age and BMI were categorized into groups, which may oversimplify the continuous nature of these factors and lead to a loss of crucial predictive information. Finally, overfitting is another possibility. Including many predictors increases the risk of overfitting, mainly when dealing with a high-dimensional dataset. Although backward elimination was used, the model's generalizability could still be compromised.

## Conclusions

Our data provides valuable insights into risk stratification in the perioperative setting. Identifying high-risk patients, such as those with advanced ASA classification or sepsis, can help clinicians target these groups for enhanced perioperative care. Moreover, logistic regression allows for quantifying these risks, assisting clinicians to understand which factors play the most significant role in adverse outcomes. To improve the model's performance and applicability, several approaches can be considered for future studies to build on this work. A more advanced model that utilizes machine learning techniques such as random forests or gradient boosting could be employed to capture non-linear relationships between variables and improve prediction accuracy. More extensive databases or more years in the analysis can be considered to address the imbalanced dataset. Likewise, techniques like oversampling of adverse events or using specialized algorithms such as synthetic minority over-sampling (SMOTE) could address the imbalance and improve the model's sensitivity to rare events. Finally, external validation on different patient populations or datasets would be necessary to assess the model's generalizability instead of validating using the same database. By leveraging a large dataset and employing advanced statistical techniques, the analysis identified key predictors and validated the robustness of the developed models. Age, BMI, ASA classification, chronic conditions like heart failure and sepsis, and smoking status emerged as strong predictors of adverse events. These predictors underscore the need for targeted interventions for at-risk patients. Likewise, the identification of risk factors for hospital resource utilization, such as underweight BMI, insulin-dependent diabetes, dialysis, and poor ASA classification, highlights the need for careful preoperative planning and post-surgical monitoring in vulnerable patient populations. By refining predictive models and enhancing preoperative risk stratification, healthcare providers can better allocate resources and tailor care to meet the needs of high-risk patients, reducing complications and improving surgical outcomes.
